# mTOR inhibitors activate PERK signaling and favor viability of gastrointestinal neuroendocrine cell lines

**DOI:** 10.18632/oncotarget.15469

**Published:** 2017-02-18

**Authors:** Patricia Freis, Julien Bollard, Justine Lebeau, Patrick Massoma, Joëlle Fauvre, Cécile Vercherat, Thomas Walter, Serge Manié, Colette Roche, Jean-Yves Scoazec, Carole Ferraro-Peyret

**Affiliations:** ^1^ University Lyon, Claude Bernard University, Cancer Research Center of Lyon, INSERM U1052, CNRS UMR5286, Faculty of Pharmacy, F-69008, Lyon, France; ^2^ Hospices Civils de Lyon, Molecular Biology of Tumors, GHE Hospital, F-69500, Bron, France; ^3^ Hospices Civils de Lyon, Digestive Oncology, Hospital E Herriot, F-69432, Lyon, France; ^4^ Institut Gustave Roussy, Biopathology, F-94800 Villejuif, France; ^5^ University Paris Sud, F-91400 Orsay, France

**Keywords:** mTOR, UPR, PERK, neuroendocrine cell lines, GI-NET

## Abstract

mTOR and Unfolded Protein Response (UPR) are two signaling pathways frequently activated in cancer cells. The mTOR pathway has been shown to be up-regulated in most gastroenteropancreatic neuroendocrine tumors. In contrast, little is known about the UPR status in neoplastic neuroendocrine cells. However, these hormone-producing cells are likely to present distinctive adaptations of this pathway, as other secretory cells. We therefore analyzed the status of the three axes of UPR and their relation to mTOR pathway in two gastrointestinal neuroendocrine tumors (GI-NET) cell lines STC-1 and GluTag. At baseline, pharmacological inducers activate the three arms of UPR: PERK, ATF6 and IRE1. Although hypoxia stimulates the PERK, ATF6 and IRE-1 pathways in both cell lines, glucose depletion activates UPR only in STC-1 cell line. Strikingly, P-p70S6K1 increases concomitantly to P-PERK and BiP in response to thapsigargin treatment, glucose depletion or hypoxia. We found that different mTOR inhibitors activate the PERK signaling pathway. To confirm that mTOR inhibition modulates PERK activation, we inhibited PERK and showed that it decreased cell viability when associated to mTOR inhibition, indicating that mTOR drives a PERK-dependent survival pathway. In conclusion, in GI-NET cell lines, UPR signaling is functional and PERK arm is induced by mTOR inhibition. These observations open up new perspectives for therapeutic strategies: the crosstalk between mTOR and UPR might contribute to the resistance to mTOR inhibitors and could be targeted by mTOR and PERK inhibitors in combination therapy.

## INTRODUCTION

Gastroenteropancreatic neuroendocrine tumors (GEP-NET) are neoplastic lesions of epithelial origin. Most of them are well-differentiated and retain the structural and functional characteristics of normal peptide-secreting endocrine cells, including the capacity to synthesize and/or secrete one or several hormones. The mechanisms of hormone synthesis and secretion in neoplastic neuroendocrine cells are similar to those operating in normal peptide-secreting endocrine cells and are controlled through the same regulatory pathways. One of these pathways is known as the unfolded protein response (UPR). In response to intrinsic or extrinsic stress inducers, UPR transiently inhibits protein synthesis and induces the production of chaperone molecules in order to restore the homeostasis of the endoplasmic reticulum (ER) and to promote cell survival [1]. The failure of this rescue mechanism results in apoptotic cell death [2]. Three ER stress transducers, controlling three distinct axes of UPR, have been identified so far. Each branch is defined by a class of transmembrane ER-resident signaling components: IRE1 (inositol requiring enzyme 1), PERK (double-stranded RNA-activated protein kinase (PKR)–like ER kinase), and ATF6 (activating transcription factor 6) [3].

Despite the importance of ER homeostasis in peptide-producing endocrine cells, little is known about UPR status and regulation in neoplastic neuroendocrine cells. UPR status has been investigated in the insulin-producing pancreatic neuroendocrine cell line INS-1 [4]. UPR activation through ATF6 has been shown to promote INS-1 survival [5, 6]. In contrast, ER stress inducers like thapsigargin, bortezomib and brefeldin A, have been shown to induce apoptosis in the human neuroendocrine cell line BON-1 and in the murine pancreatic cell line INS-1E [7, 8].

In addition to its direct impact on cell survival in response to stress, UPR might also have indirect effects. Recent results show that, in several cell types, UPR interacts with the mTORC1 (mammalian target of rapamycin complex 1) pathway [9–12]. Like UPR, the mTOR pathway, and particularly mTORC1, is involved in the response to stress and is able to promote either cell survival or apoptosis through various mechanisms. mTORC1 and PERK are co-regulated to coordinate the inhibition of protein synthesis and autophagy process when cells have to face energy depletion [13]. This co-regulation can rely on RHEB-GTP that is more available for PERK activation when mTORC1 is inactive [14]. Conversely, *Tsc1* loss in oligodendrocytes lineage leads to mTOR activation, an excessive protein translation and subsequent UPR activation through PERK–eIF2α signaling axis and Fas–JNK apoptotic pathways [15]. The UPR, and particularly PERK, is described to regulate PI3K-AKT-mTORC1 axis by activating AKT [16], increasing AMPK activity [17] or inactivating TSC2 [18]. Therefore, depending on the cell type, mTORC1 can act upstream or downstream of UPR, which can itself favor or antagonize the anabolic effects of mTORC1 [19].

The possible interplay between the UPR and the mTOR pathways might have important functional consequences in GEP-NET since the mTOR pathway is involved in their tumorigenesis. Recent sequencing studies of pancreatic and small intestinal NET showed that respectively 14 % and 33 % of cases harbor mutations in at least one gene encoding for mTOR pathway components [20, 21]. The importance of the mTOR pathway in GEP-NET is further underlined by the significant anti-tumor effects shown by the mTOR inhibitor everolimus, now used in the treatment of advanced NET [22, 23]. We therefore hypothesized that interactions between UPR and mTOR pathways might amplify the effects of mTOR on neuroendocrine cell growth and survival and might even represent a possible mechanism of resistance to mTOR inhibitors. To test this hypothesis, we decided to investigate UPR status in 2 gastrointestinal (GI)-NET cell lines and to assess their behavior and response to mTOR inhibitors using either pharmacological or metabolic stress, i.e. glucose depletion and hypoxia. We found that the three axes of UPR can be differentially activated in GI-NET cell lines, depending on the stress applied, and that mTOR inhibition is associated with an activation of PERK pathway that favors cell viability.

## RESULTS

### The three axes of the UPR are inducible by ER stress in GI-NET cell lines

**Figure 1:**
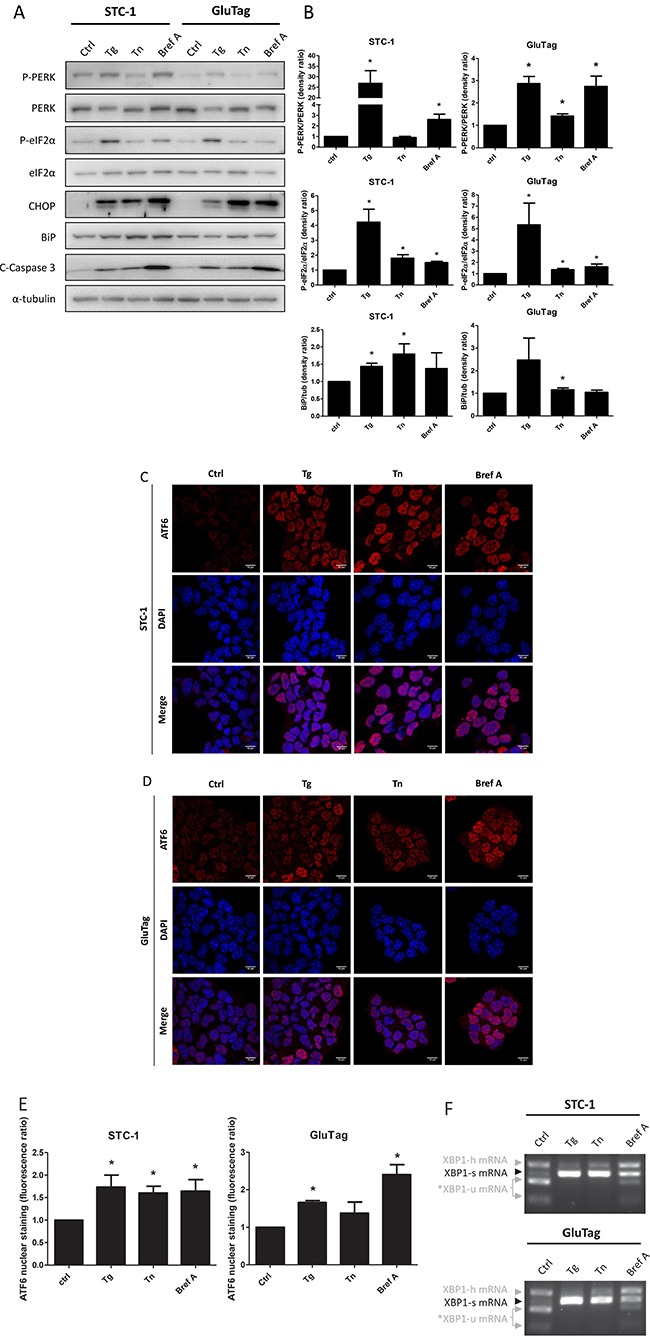
UPR status in STC-1 and GluTag cell lines and effect of UPR inducers on markers of the UPR pathways STC-1 and GluTag cells were incubated in medium (Ctrl) or ER stress-inducing agents thapsigargin (Tg, 300 nM), tunicamycin (Tn, 0.05 μg/mL) and brefeldin A (Bref A, 3 μM) for 4 h, 16 h and 8 h respectively. (**A**) Protein expression level of phosphorylated or total forms of PERK, eIF2α and CHOP, BiP and cleaved-caspase 3 (C-Caspase 3) protein expression was examined using Western Blot analysis. α-tubulin was used as internal control (**B**) Densitometric quantification of P-PERK/PERK, P-eIF2α/eIF2α and BiP/α-tubulin ratios analysis in STC-1 or GluTag cell lines (*P < 0.05 versus control). (**C**–**D**) The effect of Tg, Tn and Bref A on ATF6 nuclear localization was assessed by immunofluorescence in STC-1 cells (C) and GluTag cells (D) using anti-ATF6 antibody and Hoechst dye. Magnification ×1000. (**E**) Bar graphs obtained by quantification of ATF6 nuclear staining (*P < 0.05 versus control). (**F**) XBP1 mRNA splicing was analyzed by RT-PCR after Pst1 digestion: XBP1-h, hybrid, XBP1-u, unspliced; XBP1-s, spliced variant of XBP1; *, XBP1-u mRNA fragments after Pst1 digestion. Results are presentative of 3 independent experiments (A, C, D, F) or the mean ± S.E.M. of an experimental *n* = 3 (B, E).

As UPR has never been investigated in GI-NET, we first studied the status of the three UPR pathways, in STC-1 and GluTag cells, in basal conditions and after ER stress induction by three different mechanisms: inhibition of the sarcoplasmic/endoplasmic Ca^2+^-ATPase, blockade of N-linked glycosylation or ER to Golgi protein trafficking, using thapsigargin (Tg), tunicamycin (Tn) or brefeldin A (Bref A), respectively. As shown in Figure 1A in control conditions, activation of PERK-eIF2α axis was higher in STC-1 than in GluTag cells, as demonstrated by P-PERK and P-eIF2α. When STC-1 and GluTag cells were treated with Tg or Bref A, an activation of the PERK-eIF2α axis was observed and associated to an increased expression of the UPR pro-apoptotic target gene: CHOP. This result correlated to the cleavage of the proapoptotic factor caspase 3 (Figure 1A), indicates a functional apoptotic pathway triggered by the UPR induction. Nevertheless, after Tg treatment, PERK was less activated in GluTag cells than in STC-1 cells, with only a 3-fold increase of band density in GluTag cells compared to a 30-fold increase in STC-1 cells.

Similar amount of BiP (Figure 1A) and nuclear localization of ATF6 were observed in both cell lines in control conditions (Figure 1C and 1D). The basal nuclear ATF6 in STC-1 cell lines was confirmed using sub-fractionation (Supplementary Figure 1). STC-1 cells treated with UPR inducers showed an increase in BiP expression, which mainly reflects the activation of the ATF6 branch [24], compared to control (Figure 1A and 1B). In GluTag cells, only Tn treatment induced BiP expression (Figure 1A and 1B). Each of the three UPR inducers significantly increased the nuclear localization of ATF6 in STC-1 cells (Figure 1C and 1E), whereas only Tg and Bref A significantly increased ATF6 nuclear staining in GluTag cells (Figure 1D and 1E).

The third axis of UPR controlled by IRE-1, was investigated by monitoring the splicing of XBP1 (XBP1-s) at both RNA and protein levels (Figure 1A and 1F, Supplementary Figure 2A). This axis was activated by the three UPR-inducers, as shown by the detection of the spliced form of XBP-1 at the mRNA and the protein levels (Figure 1F and Supplementary Figure 2A, respectively).

**Figure 2:**
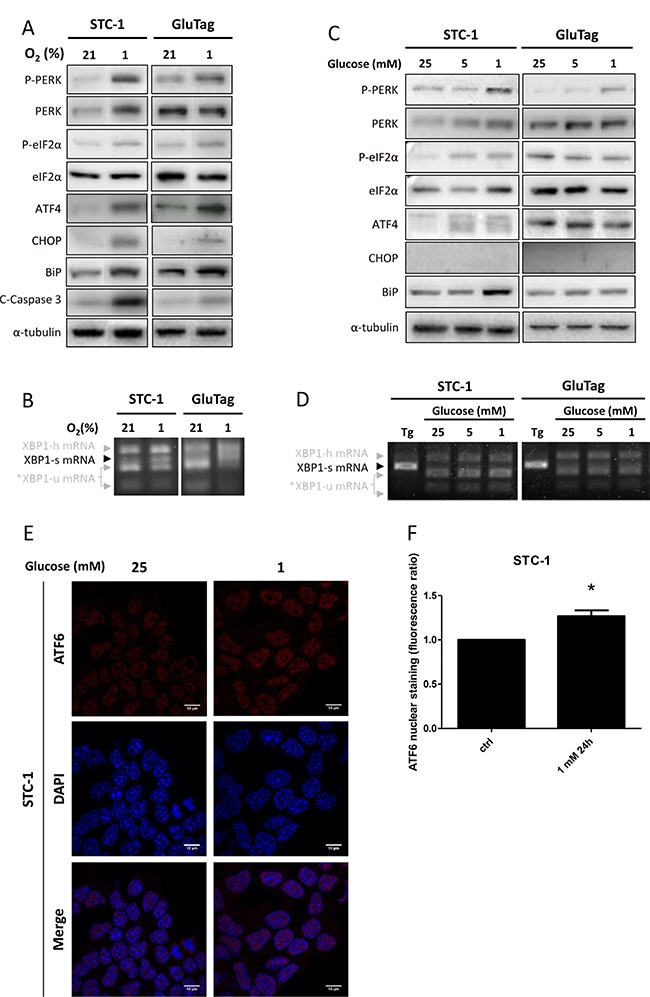
Activation of the UPR during hypoxia or glucose depletion STC-1 and GluTag cells were subjected to hypoxia (1%) or cultivated with decreasing concentration of glucose i.e. 25, 5 or 1 mM, for 24 h. (**A**) and (**C**) Protein expression level of phosphorylated and total forms of PERK, eIF2α and ATF4, CHOP, BiP and C-Caspase 3 protein expression was examined using immunoblots, during hypoxia (A) and glucose depletion (C). α-tubulin was used as internal control. Blots of Figure 2A have been performed on the same electrophoresis gel, but cut and reconstituted. (**B**) and (**D**) XBP1 mRNA splicing was analyzed by RT-PCR after Pst1 digestion: XBP1-u, unspliced; XBP1-h, hybrid; XBP1-s, spliced variant of XBP1; *, XBP1-u mRNA fragments after Pst1 digestion. (**E**) ATF6 nuclear localization was assessed in STC-1 cells using immunofluorescence with anti-ATF6 antibody and hoechst dye. Magnification x1000. Results are presentative of at least 3 independent experiments (A–E). (**F**) Bar graphs were obtained by quantification of ATF6 nuclear staining (*P < 0.05 versus control). Results are presentative of 3 independent experiments (A–E) or the mean ± S.E.M. of an experimental *n* = 3 (F).

UPR pathways were also investigated when cells were subjected to hypoxia or metabolic stress. STC-1 and GluTag cell lines were cultured in 1% O_2_ or in low glucose media (i.e. 1 or 5 mM vs 25 mM glucose) for 24 h (Figure 2).

Hypoxic conditions led to the phosphorylation of PERK and eIF2α and to the increased expression of ATF4 and CHOP in both cell lines (Figure 2A and Supplementary Figure 3A). BiP expression was also induced in both cell lines, suggesting a sustained activation of ATF6 branch (Figure 2A). Regarding the activation of IRE1 axis, hypoxia triggered the XBP1 splicing in both cell lines (Figure 2B and Supplementary Figure 2B).

In response to glucose depletion, a phosphorylation of PERK was also observed in both STC-1 and GluTag cell lines (Figure 2C). Nevertheless, the activation of PERK pathway was associated with the phosphorylation of eIF2α and the increase of ATF4 in the STC-1 cell line only. BiP expression was also induced in the two cell lines (Figure 2C, Supplementary Figure 3B). These data are supported by the accumulation of active ATF6 in the nucleus in STC-1 cells following glucose deprivation (Figure 2E and 2F). The splicing of XBP1 (mRNA or protein level) was not induced by glucose depletion in both cell lines (Figure 2C and 2D, and Supplementary Figure 3C).

Altogether, these results showed that STC-1 and GluTag cell lines were able to activate the three axes of the ER stress after treatment with UPR inducers or in response to hypoxia. After 24 h of glucose depletion, only PERK and ATF6 arms were activated in STC-1 cell line whereas such conditions did not activate the UPR in GluTag cell line.

### Induction of UPR and mTORC1 pathways are concomitant in GI-NET cell lines

As mTORC1 is known to activate ER stress in tuberous sclerosis [25] and diabetes [10, 26, 27] and to be strongly activated in GEP-NET, we investigated the activation of mTORC1 pathway on UPR activation.

**Figure 3:**
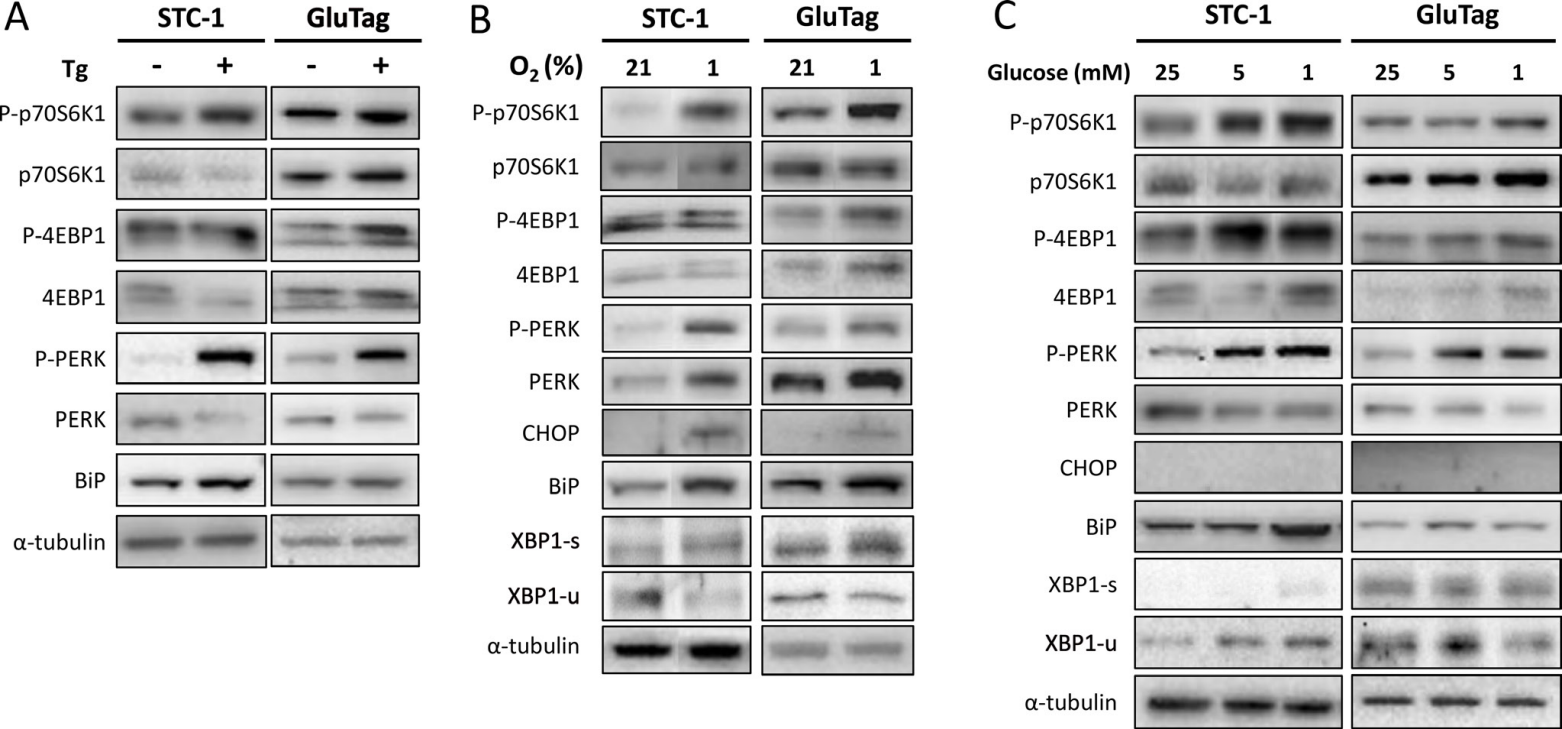
Glucose depletion or hypoxia induced a concomitant induction of UPR and mTORC1 pathways Cells were cultivated in 25 mM glucose media. (**A**) Total protein extracts prepared from STC-1 or GluTag cells treated for 4 h with 300 nM thapsigargin (Tg) were subjected to Western Blot analysis using specific antibodies either for the markers of mTOR pathway: p70S6K1 and 4E-BP1, or for UPR proteins: PERK and BiP. α-tubulin was used as internal control. (**B**) The effect of hypoxia (1% O_2_) was assessed at 24 h on the phosphorylation of p70S6K1, 4E-BP1 and PERK, CHOP, BiP, XBP1-s and XBP1-u protein expression. Blots of Figure 3B have been performed on the same electrophoresis gel, but cut and reconstituted (except for XBP1 protein). (**C**) Cells were cultivated in 25, 5 or 1 mM glucose media for 24 h. Protein extracts were used for western blotting with antibodies against the indicated proteins. α-tubulin was used as internal control. Results are representative of at least 3 experiments.

When STC-1 or GluTag cells were treated with the ER stress inducer Tg for 4h, the phosphorylation of two effectors of mTORC1: p70S6K1 and 4E-BP1, increased in both STC-1 and GluTag cells (Figure 3A). mTORC1 activation was concomitant with PERK phosphorylation in both cell lines and with an increase of BiP expression statistically significant in STC-1 cell line only (Figure 3A and Supplementary Figure 4).

After 24 h of shortage of either oxygen or glucose, comparable results were observed, with a phosphorylation of p70S6K1 and 4E-BP1, as well as UPR-related markers P-PERK, and the induction of BiP. CHOP and XBP1-s expressions were detectable only in cells cultured in 1% O_2_ but not in low glucose (Figure 3B and 3C).

Altogether, these results revealed that after 24 h of metabolic stress or hypoxia, STC-1 and GluTag cell lines concomitantly activated mTORC1 and PERK signaling pathways.

### Inhibition of mTORC1 leads to the selective induction of PERK pathway in GI-NET cell lines

**Figure 4:**
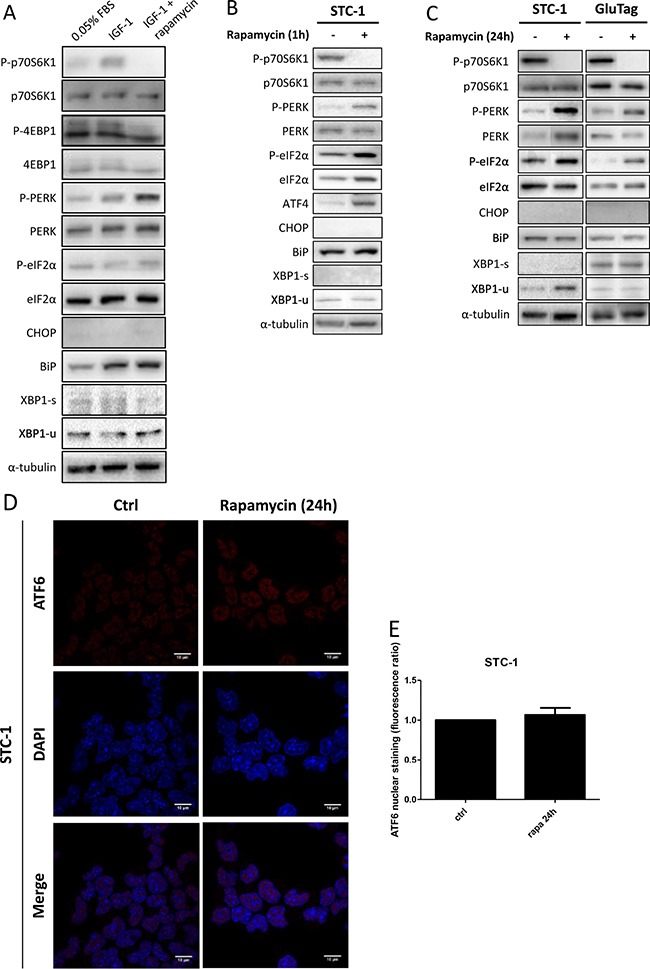
Effect of rapamycin on markers of the UPR pathways (**A**) Cells were incubated in medium alone (0.05% FBS), with 3 nM IGF-1 or with both 3 nM IGF-1 and 10 nM rapamycin (IGF-1 + rapamycin). Total protein extracts prepared from cells incubated for 24 h in those conditions were subjected to Western Blot analysis. Protein expression levels were assessed for phosphorylated or total forms of PERK, eIF2α and for CHOP, BiP, XBP1-s and XBP1-u proteins. Efficiency of rapamycin to inhibit mTORC1 pathway was also checked by immunoblot with phosphorylated and total forms of p70S6K1 and 4E-BP1. α-tubulin was used as internal control. (**B** and **C**) Cells were incubated with 10nM rapamycin for 1 h (B) or 24 h (C). Immunoblots for phosphorylated and total forms of PERK, eIF2α, p70S6K1 and 4E-BP1 or for ATF4, CHOP, BiP, XBP1-s and XBP1-u protein expression were performed. α-tubulin was used as internal control. Blots of P-p70S6K1, p70S6K1, P-PERK, PERK, and α-tubulin of Figure 4B and blots of Figure 4C have been performed on the same electrophoresis gel, but cut and reconstituted. (**D**) Cells were incubated in medium alone (Ctrl) or with 10 nM rapamycin for 24 h. Nuclear localization of ATF6 was assessed using immunofluorescence with ATF6-antibody (red) and Hoechst dye. Magnification ×1000. (**E**) Bar graphs were obtained by quantification of ATF6 nuclear staining. Results are representative of at least 3 experiments.

We wondered whether mTORC1 could modulate UPR in GI-NET cell lines or not. To address this question, in STC-1 cells, mTORC1 pathway was either activated with IGF-1 (3 nM) or inhibited using rapamycin (10 nM), for 24 h. The expression profile of UPR and mTOR pathway-related proteins was analyzed (Figure 4A). As expected, IGF-1 induced mTORC1 signaling pathway by increasing phosphorylation of p70S6K1 and 4E-BP1. UPR proteins such as P-PERK and BiP were also induced. However, CHOP expression remained undetectable. Finally, IRE-1 axis was not activated with IGF-1 treatment, as the spliced form of XBP-1 was not augmented.

The addition of rapamycin to IGF-1-treated cells suppressed mTORC1 activation, as shown by the absence of p70S6K1 phosphorylation and the decrease of 4E-BP1 phosphorylation. In this condition, we also observed a phosphorylation of PERK (Figure 4A). This activation was associated to a phosphorylation of eIF2α, whereas CHOP was not detectable.

As mTOR pathway is already activated at baseline, we studied the effect of rapamycin on UPR without adding IGF-1 to the culture media (Figure 4B–4E). After 1 h of treatment, the activation of PERK-eIF2α-ATF4 axis was observed in STC-1 cells. However CHOP was not expressed (Figure 4B). PERK activation was maintained after 24 h, in both STC-1 and GluTag cells (Figure 4C and Supplementary Figure 5A and 5B). The two other axes of UPR, IRE-1 and ATF6, were not activated by rapamycin as neither XBP1-s nor BiP expressions were modified, after 1h of treatment (Figure 4B) or from 2 to 24 h (Supplementary Figure 5A and 5B). Furthermore in STC-1 cells, the nuclear localization of ATF6 was not altered by rapamycin treatment (Figure 4D and 4E).

**Figure 5:**
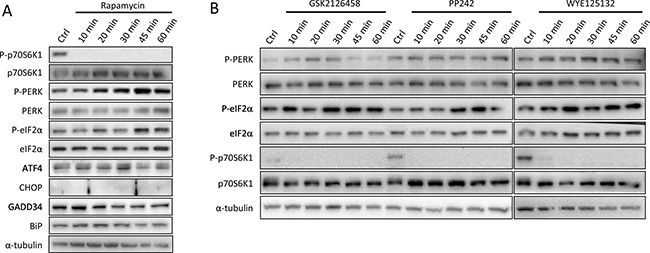
Induction of PERK pathway at early times of mTOR inhibitors treatments (**A**) Cells were cultured in medium alone (Ctrl) or with 10 nM rapamycin for 10 to 60 minutes. Protein expression levels were checked by immunoblot for phosphorylated and total forms of p70S6K1, PERK, eIF2α, and for ATF4, CHOP, GADD34 and BiP protein expression. (**B**) Effect of mTOR inhibitors on PERK activation. Cells were cultured in medium alone (Ctrl) or with specific inhibitors of mTOR: GSK2126458 (10 nM), PP242 (1 μM) or WYE125132 (100 nM) for 10 to 60 minutes. Total protein extracts were subjected to Western Blot analysis using specific antibodies against the indicated protein. α-tubulin was used as internal control. Results are representative of 3 experiments.

We then monitored UPR status following short period of rapamycin exposure. A quick phosphorylation of PERK and a consecutive phosphorylation of eIF2α occurred after 20 minutes (Figure 5A). The expression of ATF4 was increased after 30 min of rapamycin treatment and then decreased, probably due to the action of negative feedback loops. The exposure to rapamycin, as for the other duration of treatment, did not lead to the increase of CHOP, BiP or GADD34 expression (Figure 5A).

Three other mTOR inhibitors, namely GSK2126458 (100 nM), PP242 (1 μM) or WYE125132 (100 nM), were tested (Figure 5B). All the three drugs completely inhibited p70S6K1 phosphorylation and rapidly induced PERK and eIF2α phosphorylation, i.e. 10 min to 30 min of treatment. PERK remained phosphorylated up to 60 min with PP242 and WYE125132 treatments, whereas it decreased after 30 min with GSK2126458 treatment.

Altogether, these results showed that mTORC1 inhibition mostly impact on one axis of UPR. Indeed, when treated with mTORC1 inhibitors, GI-NET cell lines activated PERK pathway, whereas IRE-1 axis and BiP status were not modified.

### The activation of PERK pathway with rapamycin increases cell viability

As PERK pathway determines cell fate, depending on the duration and the intensity of its activation [28], we wonder if its activation after mTOR inhibition can modify cell viability.

**Figure 6:**
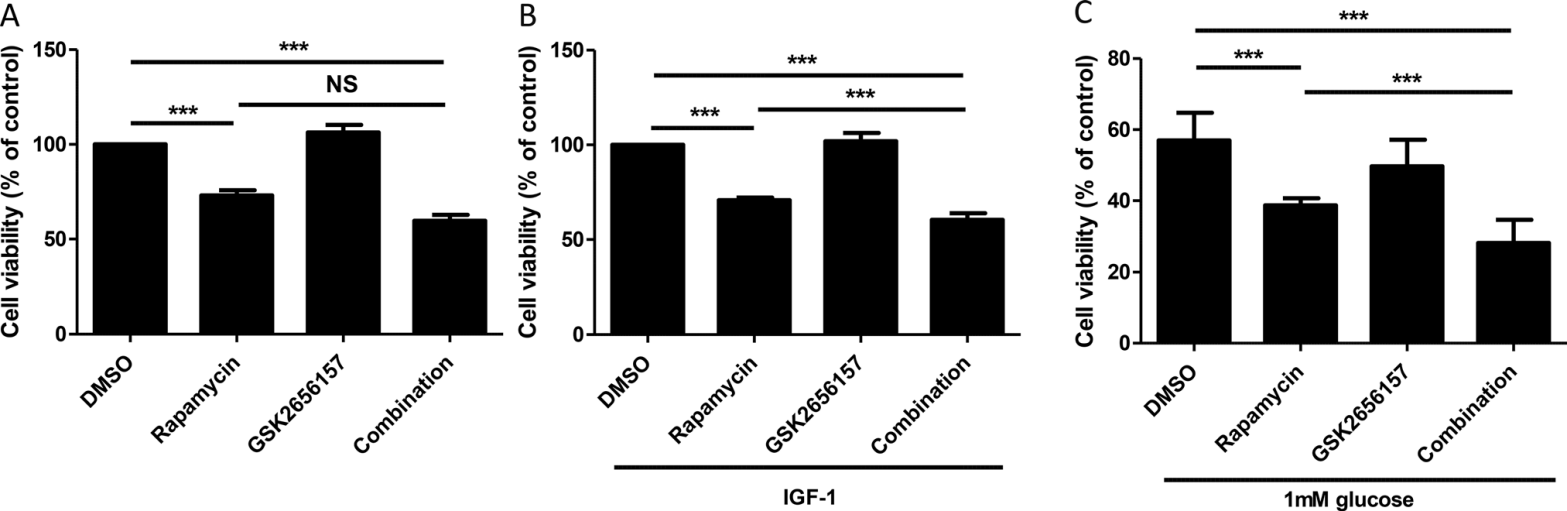
Effect of PERK Inhibitor GSK2656157 and rapamycin on cell proliferation, estimated by measuring the number of viable cells (**A**) STC-1 cells were incubated with rapamycin (10 nM) or GSK2656157 (100 nM) or both for 24 hours, then viability was assessed using MTT test. (**B**) Cells were cultivated in 0.05% FBS overnight then incubated with exogenous IGF-1 at 3 nM and treated with rapamycin (10 nM) or GSK2656157 (100 nM) as described in A. (**C**) Cells were cultivated in 1 mM glucose medium for 24 h then treated with rapamycin (10 nM) or GSK2656157 (100 nM) as described in A. The graph shows the mean of at least 3 independent experiments ± S.E.M. Statistical analysis was performed using Holm-Sidak Test, ^**^*P < 0.001.

We therefore treated STC-1 cell line either with rapamycin or with an inhibitor of PERK phosphorylation: GSK2656157 or both drugs for 24 h. mTOR inhibition by rapamycin significantly decreased cell viability by 27% after 24 h of treatment (*p* < 0.001) (Figure 6A). PERK inhibitor GSK2656157 alone had no significant effect as well as combined with rapamycin (Figure 6A). However, in GI-NET, mTOR is overactivated [29], so to mimic this tumoral feature we evaluated the efficacy of the combination in two context of mTORC1 activation, i.e. IGF-1 treatment and glucose depletion. When STC-1 cells proliferation and mTOR pathway were stimulated by the growth factor IGF-1, rapamycin significantly decreased cell viability (30%, *p* < 0.001). GSK2656157 had no effect on cell viability, whereas the combination of both drugs decreased significantly cell viability compared to rapamycin condition (40% *vs* 30%, *p* < 0.001, Figure 6B and Supplementary Figure 6). When cells were cultured in 1 mM glucose, rapamycin alone induced a significant fall of cell viability (31%, *p* < 0.001) whereas GSK2656157 had still no effect (Figure 6C). The combination of both drugs significantly decreased cell viability compared to rapamycin alone (50% *vs* 31%, *p* < 0.001).

All these data show that the co-inhibition of the mTORC1 and PERK pathways decreases cell viability in a context of mTORC1 activation.

## DISCUSSION

We here demonstrated that neoplastic GI-NET cells, subjected to stressful conditions such as hypoxia or glucose depletion, are able to simultaneously activate mTOR and UPR pathways. We also showed that mTOR inhibition by therapeutic agents resulted in the activation of the PERK axis of UPR.

UPR is known to play various physiological roles in both normal and neoplastic peptide-producing endocrine cells. Several concurrent studies on pancreatic cell function demonstrated that UPR is necessary for cell survival, hormone synthesis and secretion in β-pancreatic cells or insulinoma-derived cell lines [4, 30]. Recently, Hassler *et al*. [31] also showed that secretion of insulin depends on the activation of XBP1s–dependent secretive genes. Indeed, IRE1 is a determinant pathway [32] for pancreatic β-cell survival, while PERK and its downstream effector CHOP are associated to β-cell death [33]. The activation of UPR has not been described in GI-NET cell lines, nor in human GI-NET yet. As well-differentiated neuroendocrine tumors still synthetize and secrete a large amount of neuroendocrine peptides [34], we might extrapolate that neuroendocrine tumor cells may activate UPR to maintain ER homeostasis and prevent the accumulation of unfolded proteins.

In the present study, we observed the expression of PERK, ATF6 and IRE1 axes components in two cell lines derived from GI-NET, STC-1 and GluTag, both known to be highly secretory cells [35, 36]. Nonetheless, the activation of PERK axis did not lead to the expression of CHOP, a pro-apoptotic protein downstream of PERK axis, suggesting that the activation of PERK pathway is too weak to induce apoptotic pathway. When we assessed the effect of three different UPR inducers, Tg, Tn or Bref A, IRE-1 and ATF6 axes were strongly induced by the three drugs. The effect of these drugs on PERK axis varied, depending on the drug and cell types. In both cell lines, Tg and BrefA induced PERK axis, while Tn significantly induced PERK axis in GluTag cells only. These differences can be explained by the three distinct mechanisms of action of these drugs. Finally, pharmacological induction of ER stress resulted into cell apoptosis, as both cell lines strongly expressed pro-apoptotic CHOP and cleaved-caspase 3 proteins when treated with Tg, Tn or Bref A, suggesting that a high level of ER stress led to cell death.

During tumor growth, two types of stress are known to induce UPR, namely glucose depletion and hypoxia. These stressful factors force tumor to adapt in order to survive until the organization of neovascularization provides nutrient and oxygen supplies. When exposed to hypoxia, both cell lines activated the three axes of UPR, which led to increased CHOP expression and consequent caspase 3 cleavage. In contrast, after 24 h of glucose depletion, only STC-1 cell line activated PERK axis, without inducing CHOP. These results suggest that GI-NET cell lines are more sensitive to oxygen deprivation than to glucose decrease. PERK appears to be a key pathway in neuroendocrine cells. PERK is known to be pivotal for cell adaptation to ER stress, promoting either survival or apoptosis. Indeed, on one hand, in order to re-establish ER homeostasis, PERK is able to: (a) decrease cellular anabolic requirements by inhibiting cell proliferation thanks to cyclin D1 downregulation, (b) inhibit Cap-dependent translation protein and therefore decrease protein load within the ER, (c) maintain redox homeostasis via Nrf2 transcription and (d) upregulate the transcription factor ATF4 in order to induce the expression of prosurvival genes. On the other hand, PERK may promote apoptosis after prolonged or chronic activation as the permanent nuclear localization of ATF4 induces the expression of the proapoptotic factor CHOP [28].

mTOR and UPR have been shown to act coordinately in a number of biological processes [19]. In our study, when we investigated mTORC1 expression after metabolic stress, we observed a concomitant activation of mTORC1, PERK, BiP and XBP1-s in hypoxia conditions, and a concomitant activation of mTORC1, PERK and BiP in glucose depletion conditions. STC-1-cells treatment with IGF-1, which activate mTORC1 pathway, resulted in a concomitant up-regulation of mTORC1, PERK and BiP. To assess whether PERK activation and BiP expression depend on mTORC1 activation, cells were treated with rapamycin. Unexpectedly, addition of rapamycin in STC-1 cells did not reversed PERK phosphorylation nor BiP expression. In contrast, in both cell lines, rapamycin treatment strongly and quickly up-regulated PERK axis, i.e. after 20 minutes of treatment. This was confirmed using other mTORC1 inhibitors which do not interfere with the stability of mTORC1 but are ATP-competitive inhibitors. Tyagy et al. recently described in HEK293 cell line that RHEB, an essential upstream activator of mTORC1, is more available when mTORC1 is inhibited and could thereby promote PERK activation [14]. mTORC2 can also regulate PERK/eIF2α axis, as it was recently showed in TSC2^−/−^ MEF by Tenkerian *et al*. [11, 37]. Further investigations are required to understand whether such mechanisms are implicated in the activation of PERK when GI-NET cell lines are treated with mTORC1 inhibitors. We demonstrated that the combination of both mTOR inhibitor and PERK inhibitor decreased significantly cell viability, compared to mTOR inhibitor alone. This suggests that activation of PERK pathway with mTOR inhibitor is prosurvival. Those results suggest that targeting PERK axis activation could enhance effectiveness of mTOR inhibitor treatment in patients. More studies are needed to confirm these results in *in vivo* models of GEP NET.

As UPR can be activated and modulated in GI-NET cell lines, we also wonder whether it could behave as a therapeutic target. This option has been scarcely studied until now. Bortezomib, sanguinarine and brefeldin A have been described to decrease cell viability in the human pancreatic neuroendocrine BON-1 cell line [7]. Only one clinical trial has evaluated the effect of the UPR inducer bortezomib in 16 patients with various well-differentiated neuroendocrine tumors [38]. In this study, bortezomib did not induce any objective response but any definitive interpretation is precluded by the very small number of patients and the unknown profile of UPR or mTOR activation in treated tumors. Further studies are needed to analyze the UPR status and also to evaluate the effects of UPR inducers in GEP-NET.

## MATERIALS AND METHODS

### Cell lines

The STC-1 cell line, a gift of G. Rindi (Department of Pathology and Laboratory Medicine, Roma, Italy) and the GluTag cell line, a gift of D.Drucker (Department of Medicine, Mt Sinai Hospital, Toronto, Ont., Canada) are derived from neuroendocrine intestinal tumors developed in transgenic mice. Both cell lines retain the capacity to synthesize and secrete peptidic hormones and neuromediators [35, 36]. The MCF-7 cell line was purchased from the ATCC. Cells were routinely cultured in Dulbecco's modified Eagle's medium (DMEM) supplemented with 5% for STC-1 and MCF-7 cells or 10% fetal bovine serum (FBS) for GluTag cells, 2 mM glutamine and antibiotics (100UI/ml penicillin, 100 μg/ml streptomycin), in 5% CO_2_ and 37°C conditions.

### Glucose depletion

Cells were seeded in 6-well plates (for protein extraction) or in 12-well plates (for immunofluorescence studies) and maintained 48 hours under normal culture conditions, i.e. 25 mM glucose concentration, 5% or 10% FBS, 5 % CO_2_, 21% O_2_. Glucose depletion was obtained by removing 25 mM glucose medium and adding fresh medium containing 5 mM or 1 mM glucose, for 24 h. Experiments were stopped by removing media; then, 6-well plates were washed 2 times with cold PBS for protein extraction, while 12-well plates were fixed with paraformaldehyde 4% for immunostaining.

### Hypoxia

Cells were seeded in 6-well plates in normal culture conditions. After 48 h, cells were transferred to 1% O_2_ environment, at 37°C and 5 % CO_2_ for 24 h.

### Cell proliferation assay

Cells were seeded at a density of 2000 cells per well in 96-well plates and maintained 72 h in normal culture conditions. The medium was then replaced by 5% FBS-containing DMEM medium with drugs (rapamycin or GSK2656157) alone or in combination for 24 h. A 10 uL sample of 3-(4,5-dimethylthiazol-2-yl)2,5-diphenyltetrazolium bromide (MTT) solution (5 mg/ml) was added to each well, and the plates were incubated at 37°C for 2 h. If cell proliferation was stimulated by IGF-1, the medium was firstly replaced by 0.05% FBS-containing DMEM medium with IGF-I for 24 h before drugs were added. The supernatant was discarded, and 100 uL of DMSO was added to dissolve formazan crystals, generating a blue-purple color. The absorbance was measured at 540 nm.

### Reagents

Murine recombinant insulin-like growth factor-1 (IGF-1) was purchased from PeproTech (Rocky Hill, NJ). Thapsigargin was obtained from Applichem (St Louis, MO), tunicamycin from Sigma (St Louis, MO) and brefeldin A from TOKU-E (Bellingham, WA). mTOR and PERK inhibitors were purchased from LC Labs (Woburn, MA) (rapamycin), Selleckchem (Houston, TX) (WYE125132, GSK2656157), Abcam (Cambridge, UK) (PP242) and from GlaskoSmith and Kline (Middlesex, UK) (GSK2126458). Antibodies against phospho-PERK (Thr980) (No.3179), PERK (No. 3192), phospho-eIF2α (Ser51) (No. 3597), eIF2α (No.2103), phospho-p70S6K1^thr389^ (No. 9234), p70S6K1 (No. 9202) and cleaved-caspase3 (No. 9664) were purchased from Cell Signaling Technology (Beverly, MA). Phospho-4E-BP1 (phosphorylation on Thr45; ab68187), and 4E-BP1 (ab2606) were from Abcam (Cambridge, UK). ATF6 clone 70B1413.1 were from Abcam (Cambridge, UK; ab11909) and Novus Biological (Littleton, CO; NBP1-40256). Antibodies against CHOP (SC-575), ATF4 (SC-200), GADD34 (SC-8327) and XBP1 (SC-7160) came from SantaCruz Biotechnology (Santa Cruz, CA) and BiP (610978) from BD Laboratories^™^ (Franklin Lakes, NJ). Antibody against α-tubulin was from Sigma-Aldrich (St. Louis, MO).

### Protein extraction and Western blot analysis

After glucose withdrawal, hypoxia or drug treatments for the given times, cells were washed 2 times with cold PBS and lysed in cold solubilization buffer (pH 8) containing 150 mM NaCl, 50 mM Tris-base, 2 mM EDTA, 0.5% deoxycholate, 0.1% SDS, 1% NP40, 2 mM orthovanadate, and 20 mM NaF. Cell lysates were centrifuged at 13,000 rpm for 20 min at 4°C and the supernatant was stored at –80°C. Protein yield was quantified using the Bradford protein assay kit. The absorbance was read after 10 min of incubation at 595 nm. Total protein lysates (20 μg) were then separated by electrophoresis on a 10% SDS-PAGE gel and transferred to a polyvinylidene difluoride membrane (PVDF) (Millipore, Billerica, MA). Saturation was achieved in a 0.1% Tween 20 Tris-buffered saline solution containing 5% non-fat dry milk for 1 h. Membranes were hybridized with primary antibodies overnight at 4°C, washed and incubated with the corresponding immunoperoxidase-conjugated secondary antibody (Jackson ImmunoResearch Laboratories; Beckman Coulter France, Roissy, France) for 1 h at room temperature. Immunodetection was performed using electrochemiluminescence (ECL Western Blotting Detection System; Covalab, Villeurbanne, France or Luminata Crescendo, Millipore, Billerica, MA) and acquired with Chemi-Doc XR5 machine (Bio-Rad, Marnes la Coquette, France). Quantity One software were used. To avoid cross-detection between phosphorylated and total forms of the protein, membranes were stripped.

### RNA extraction and XBP1 splicing assessment

Total RNA was isolated from cells with the Nucleospin RNA kit (Macherey-Nagel, Hoerdt, France). Complementary DNA was synthesized with Superscript II (Invitrogen, Carslbad, CA) with oligo(dT) primers (Invitrogen, Carslbad, CA). The active form of XBP1 is its spliced form. The unspliced form of XBP1 possesses a Pst1 restriction site, which is not present on the spliced form. The level of spliced XBP1 was assessed by amplifying its cDNA using a primer pair encompassing the missing sequences in XBP1s (5′-GAACCAGGAGTTAAGAACACG-3′ and 5′-AGGCAACAGTGTCAGAGTCC-3′) and performing a subsequent enzymatic digestion targeting the Pst-1 site. The digest was then run on a 2.5% agarose gel. The inactive/spliced form resulted in two small fragments, following digestion with Pst1 (New England Biolabs, Ipswich, MA), while the active/spliced form remained undigested. A fourth band was also obtained and corresponded to a hybrid (h) between the unspliced and spliced ssDNA formed during the PCR.

### Immunofluorescence staining

After glucose depletion or drug treatments for the given times, cells were fixed with paraformaldehyde 4%, washed 3 times with cold phosphate buffered saline (PBS) and permeabilized using PBS 0.1% Triton. Unspecific sites were blocked with 4% bovine serum albumin (BSA) diluted in PBS containing 0.1% Triton. Primary antibody was then added overnight at 4°C, in PBS 0.1% Triton containing 2% BSA. Cells were washed 3 times with PBS and incubated with the corresponding fluorescein-conjugated secondary antibody for 1h, diluted in PBS 0.1% Triton, 2% BSA. The nucleus dye Hoechst was added for 5 min, then cells were washed 3 times. Slides were observed using confocal microscope Zeiss780 and analyzed with ZEN microscope and imaging software. Nuclear signal quantification was performed using ImageJ software. All experiments were performed at least 3 times.

### siRNA transfection

siRNA oligonucleotides duplexes targeting mouse ATF6 (ON-TARGETplus SMARTpool siRNA ATF6) ON-TARGETplus or non-targeting siRNAs were purchased from Dharmacon (Lafayette, CO). siRNA were resuspended in 1× siRNA buffer (Dharmacon). After 48 h of incubation in normal growth condition, cells were transfected with the siRNA (30 nM) using Interferin reagent purchased from Polyplus (Illkirch, France) in Optimem medium. After 24 h of incubation with the siRNA, media was aspirated gently and replaced with normal growth culture medium containing no siRNA. After 24 h and 48 h, transfection were stopped by washing cells 2 times with ice cold PBS. Subcellular fractionation was directly performed.

### Subcellular fractionation

Cells were washed with PBS, harvested by trypsin-EDTA and washed twice with PBS to remove traces of trypsin and growth medium. Pellets were lysed in buffer A containing 10 mM HEPES, pH 7.9, 1.5 mM MgCl_2_, 10 mM KCl, 0.5 mM DTT and phosphatases inhibitors NaF, Na_3_VO_4_ and beta-glycerophosphate, and proteases inhibitors, during 5 minutes on ice. Thereafter, NP-40 was added at a final concentration of 0.5% (v/v) for 10 minutes on ice. Supernatants were collected by centrifugation (3000 rpm 10 minutes at 4°C) and represent the cytoplasmic fraction. Supernatants were stored at −80°C. Nuclear pellets were washed 2 times using Buffer A as described above with NP-40 0.5%. Nuclear pellets were lysed in RIPA buffer containing 50 mM TRIS-HCl pH 8, 150 mM NaCl, NP-40 1%, deoxycholate sodium 0.5%, SDS 0.1%, phosphatases and proteases inhibitors. After vigorous vortexing, nuclear pellets were incubated 30 minutes on ice, then centrifuged at 3000 rpm for 30 minutes at 4°C. The supernatants, representing nuclear fraction, were collected and stored at −80°C.

### Statistical analysis

Results are presented as mean ± S.E.M. Significant differences were analyzed using Sigma Plot, Mann-Whitney test, *t*-tests or Holm-Sidak ANOVA test were performed. *P* < 0.05 (*) or *P* < 0.001 (***) were required for statistical significance, respectively.

## SUPPLEMENTARY MATERIALS FIGURES AND TABLES


