# Supplementary material for: mTOR inhibitors activate PERK signaling and favor viability of gastrointestinal neuroendocrine cell lines
Source: Oncotarget. 2017 Feb 18;8(13):20974–87. doi: 10.18632/oncotarget.15469 (PMC5400559; doi:10.18632/oncotarget.15469)
Supplement: Supplementary file 1 [file oncotarget-08-20974-s001.pdf]

mTOR inhibitors activate PERK signaling and favor viability of gastrointestinal neuroendocrine cell lines

Supplementary Materials

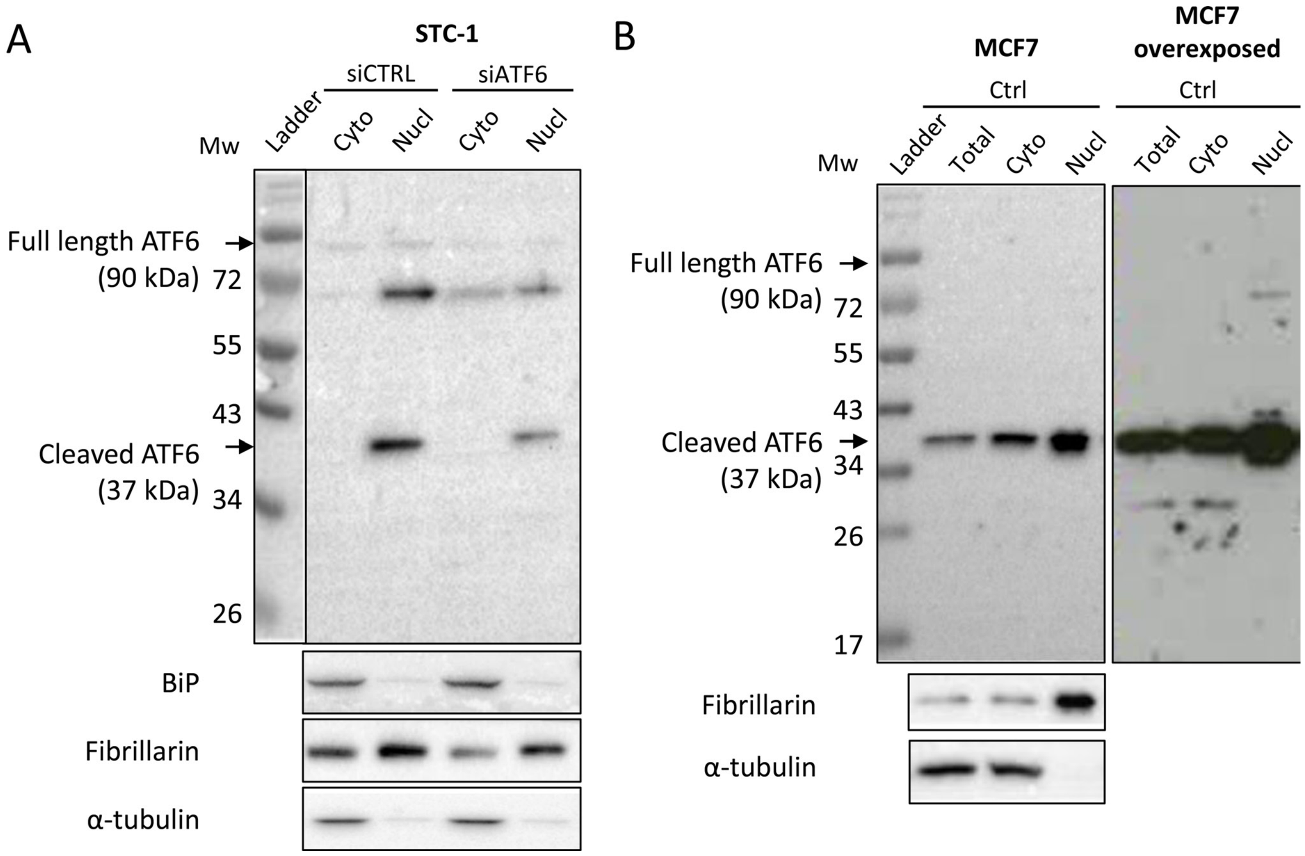

Supplementary Figure 1: ATF6 localization in STC-1 and MCF7 cells. (**A**) STC-1 cells were transfected using siRNA oligonucleotides duplexes targeting mouse ATF6 siRNA ATF6 or siCTRL (30 nM). After 48 h subcellular fractionation was performed. ATF6 protein expression in cytoplasmic and nuclear fractions were studied by western blot analysis. BiP was used as endoplasmic reticulum internal control, fibrillarin was used as nucleic internal control and α-tubulin was used as total and cytoplasmic internal control. (**B**) ATF6 protein expression in total, cytoplasmic and nuclear cell lysates of MCF7 cell line. Subcellular fractionation was performed as described above. Fibrillarin was used as nucleic internal control and α-tubulin was used as total and cytoplasmic internal control. Results are representative of 3 experiments.

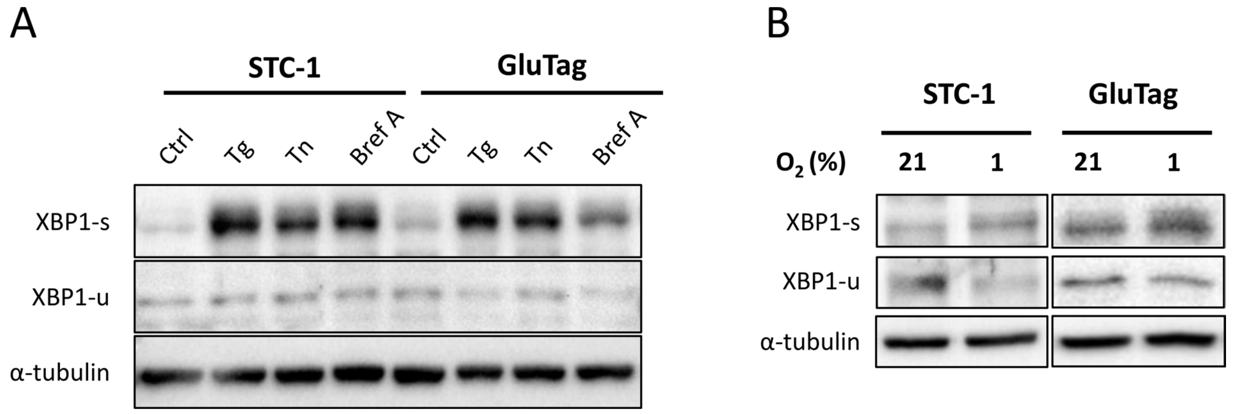

Supplementary Figure 2: Effect of UPR inducers and hypoxia on the expression of XBP1-spliced and unspliced proteins. (**A**) STC-1 and GluTag cells were incubated in medium (Ctrl) or ER stress-inducing agents thapsigargin (Tg, 300 nM), tunicamycin (Tn, 0.05 µg/mL) and brefeldin A (Bref A, 3 µM) for 4 h, 16 h and 8 h respectively. (**B**) STC-1 and GluTag cells were subjected to hypoxia for 24 h. (B) Protein expression level of XBP1 spliced or unspliced was examined using Western Blot analysis. α-tubulin was used as internal control. Results are representative of at least 3 experiments.

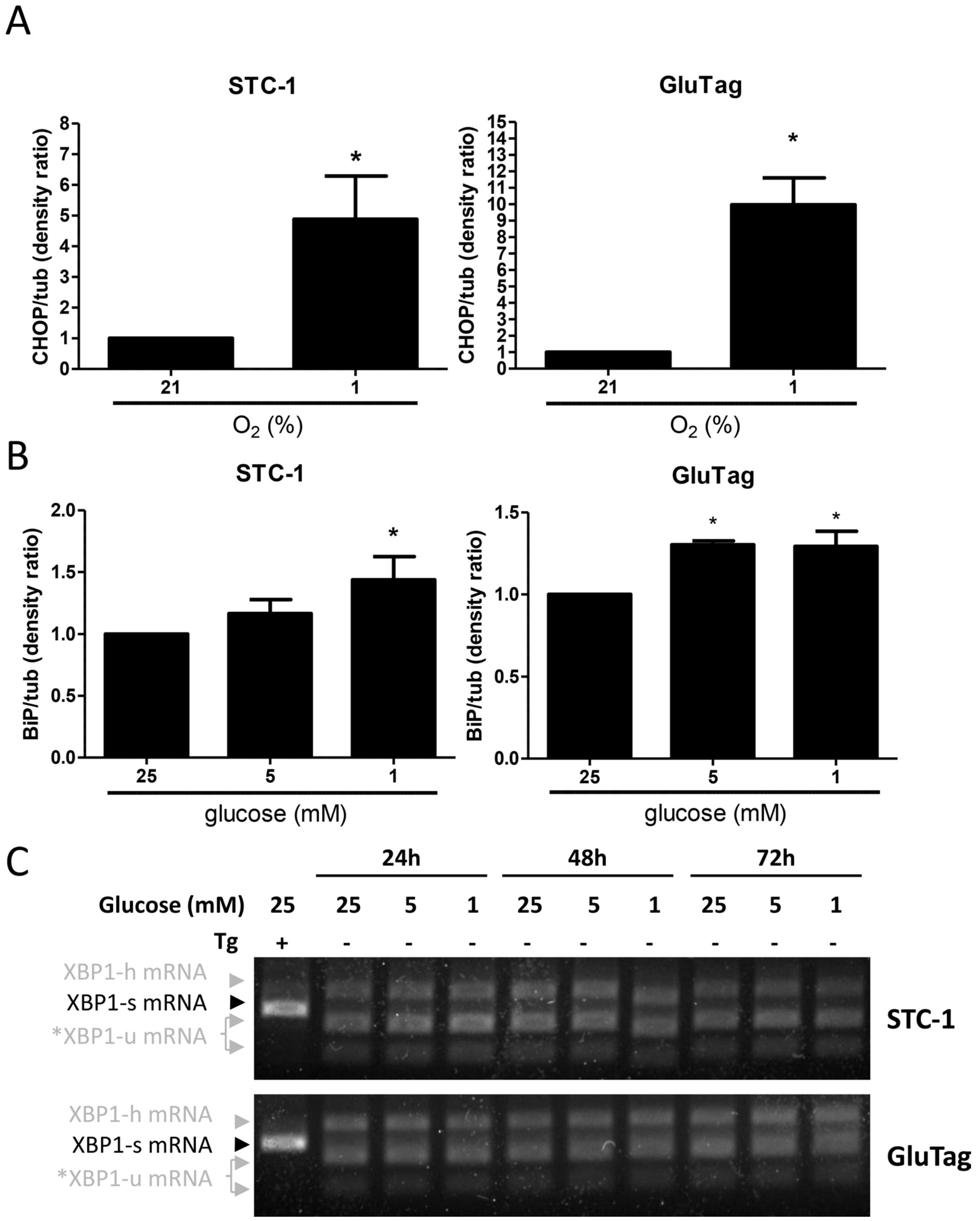

Supplementary Figure 3: Activation of the UPR during hypoxia or glucose depletion in STC-1 and GluTag cell lines. (**A**) Densitometric quantitation of CHOP/α-tubulin ratios analysis in STC-1 or GluTag cell lines submitted for 24 h to normoxia or hypoxia. Data are presented as mean ± SEM of an experimental $n = 3$ (*$P < 0.05$ versus control). (**B**) Densitometric quantitation of BiP/α-tubulin ratios analysis in STC-1 or GluTag cell lines cultured in various concentration of glucose for 24 h. Data are presented as mean ± SEM of an experimental $n = 3$ (*$P < 0.05$ versus control). (**C**) STC-1 or GluTag cells were cultivated with decreasing concentration of glucose i.e. 25, 5 or 1 mM, for 24 h, 48 h and 72 h. XBP1 mRNA splicing was analyzed by RT-PCR after Pst1 digestion: XBP1-u, unspliced; XBP1-h, hybrid; XBP1-s, spliced variant of XBP1; *, XBP1-u mRNA fragments after Pst1 digestion. Tg-treated cells were used as positive control. Results are representative of at least 3 experiments.

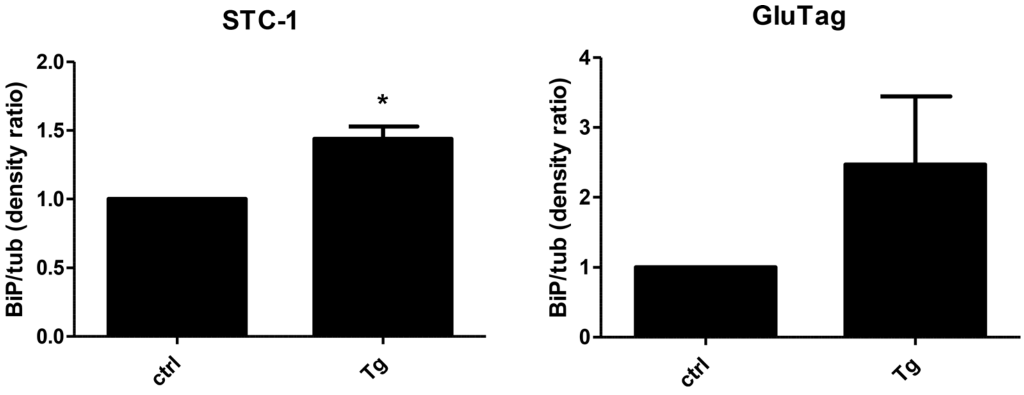

Supplementary Figure 4: Thapsigargin treatment effect on BiP expression. Densitometric quantification of BiP/α-tubulin ratios analysis in STC-1 or GluTag cell lines treated or not with Tg for 4 h Data are presented as mean ± SEM of an experimental $n = 3$ (*$P < 0.05$ versus control).

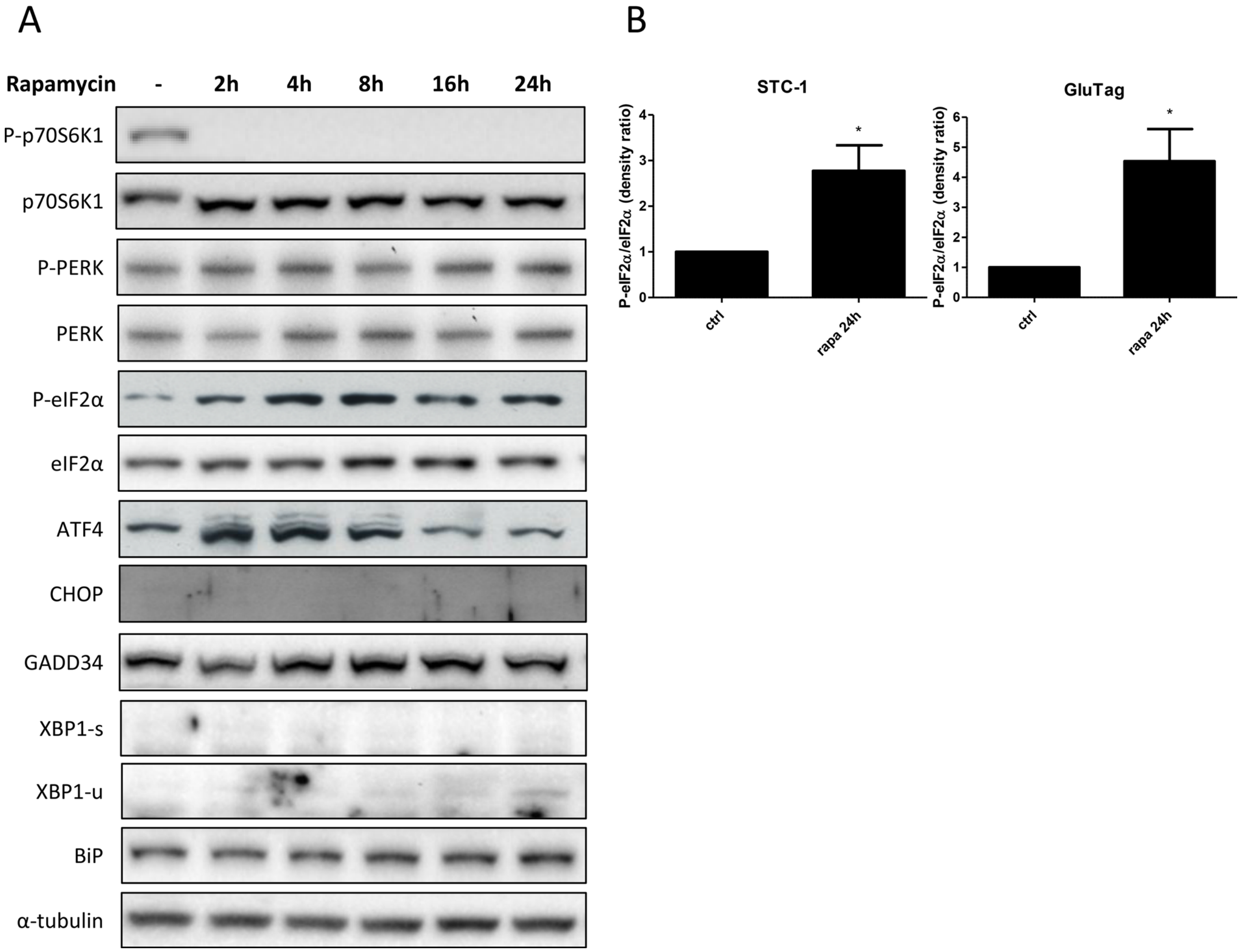

Supplementary Figure 5: Effect of rapamycin on markers of the UPR pathways. (**A**) STC-1 cells were incubated with 10 nM rapamycin for 2 h to 24 h. Total protein extracts were subjected to Western Blot analysis. Protein expression levels were assessed for phosphorylated or total forms of p70S6K1, 4E-BP1, PERK, eIF2α and for ATF4, CHOP, GADD34, BiP, XBP1-s and XBP1-u proteins. α-tubulin was used as internal control. Results are representative of at least 3 experiments. (**B**) Densitometric quantification of P-eIF2α/eIF2α ratio in STC-1 or GluTag cell lines treated or not with rapamycin for 24 h. Data are presented as mean ± SEM of an experimental $n = 3$ (*$P < 0.05$ versus control).

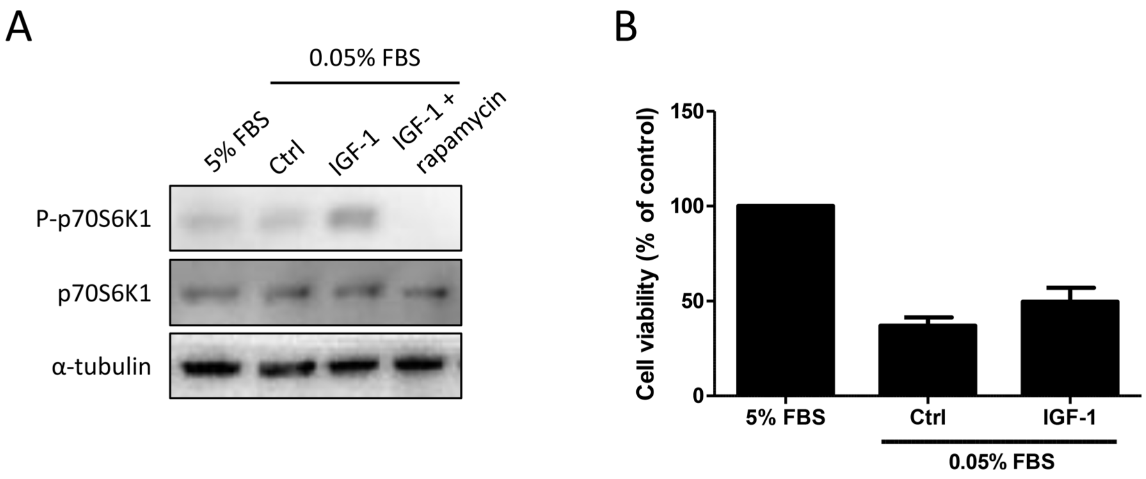

Supplementary Figure 6: Effect of serum deprivation and IGF1 activation on p70S6K1 activation and cell viability. STC-1 cells were submitted to serum deprivation (0.05% FBS) overnight before treating cells with 3 nM IGF-1 or with both 3 nM IGF-1 and 10nM rapamycin (IGF-1 + rapamycin). Usual culture medium was used as control (5% FBS). (**A**) Total protein extracts were subjected to Western Blot analysis. Protein expression levels were assessed for phosphorylated or total forms of p70S6K1. α-tubulin was used as internal control. Results are representative of at least 3 experiments. (**B**) Cell viability was assessed using MTT test over the last 2 hours of treament. The graph shows the mean of at least 3 independent experiments ± SEM
